# Supplementary material for: The Importance of Thermal Treatment on Wet-Kneaded Silica–Magnesia Catalyst and Lebedev Ethanol-to-Butadiene Process
Source: Nanomaterials (Basel). 2021 Feb 26;11(3):579. doi: 10.3390/nano11030579 (PMC7996789; doi:10.3390/nano11030579)
Supplement: Supplementary file 1 [file nanomaterials-11-00579-s001.pdf]

nanomaterials

Supplementary information

Article

The Importance of Thermal Treatment on Wet–Kneaded Silica–Magnesia Catalyst and Lebedev Ethanol–to–Butadiene Process

Sang–Ho Chung [1,*], Adrian Ramirez [2], Tuiana Shoinkhorova [2], Ildar Mukhambetov [1], Edy Abou–Hamad [3], Selevedin Telalovic [2], Jorge Gascon [2] and Javier Ruiz–Martinez [1,*]

[1] KAUST Catalysis Center, King Abdullah University of Science and Technology, Catalysis, Nanomaterials, and Spectroscopy (CNS), Thuwal 23955, Saudi Arabia; ildar.mukhambetov@kaust.edu.sa
[2] KAUST Catalysis Center, King Abdullah University of Science and Technology, Advanced Catalytic Materials (ACM), Thuwal 23955, Saudi Arabia; adrian.galilea@kaust.edu.sa (A.R.); tuiana.shoinkhorova@kaust.edu.sa (T.S.); selvedin.telalovic@kaust.edu.sa (S.T.); jorge.gascon@kaust.edu.sa (J.G.)
[3] KAUST Core Labs, King Abdullah University of Science and Technology, Thuwal 23955, Saudi Arabia; edy.abouhamad@kaust.edu.sa
* Correspondence: sangho.chung@kaust.edu.sa (S.–H.C.); javier.ruizmartinez@kaust.edu.sa (J.R.M.); Tel.: +966-(0)12-808-4530 (J.R.M.)

Citation: Chung, S.-H.; Ramirez, A.; Shoinkhorova, T.; Mukhambetov, I.; Abou–Hamad, E.; Telaovic. S.; Gascon, J; Ruiz-Martínez, J. The Importance of Thermal Treatment on Wet-Kneaded Silica–Magnesia Catalyst and Lebedev Ethanol-to-Butadiene Process. *Nanomaterials* **2021**, *11*, 579. https://doi.org/10.3390/nano11030579

Academic Editor: Juan Carlos Serrano-Ruiz

Received: 31 December 2020
Accepted: 11 February 2021
Published: 26 February 2021

Publisher's Note: MDPI stays neutral with regard to jurisdictional claims in published maps and institutional affiliations.

Copyright: © 2021 by the authors. Licensee MDPI, Basel, Switzerland. This article is an open access article distributed under the terms and conditions of the Creative Commons Attribution (CC BY) license (http://creativecommons.org/licenses/by/4.0/).

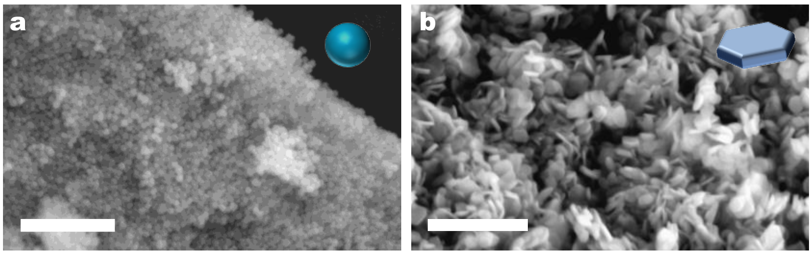

Figure S1. SEM images of wet–kneading precursors for SiO_2–MgO catalyst: SiO_2 (**a**) and MgO (**b**). The inset figures represent the schematic models of each nanoparticles. The scale bar represents 500 nm.

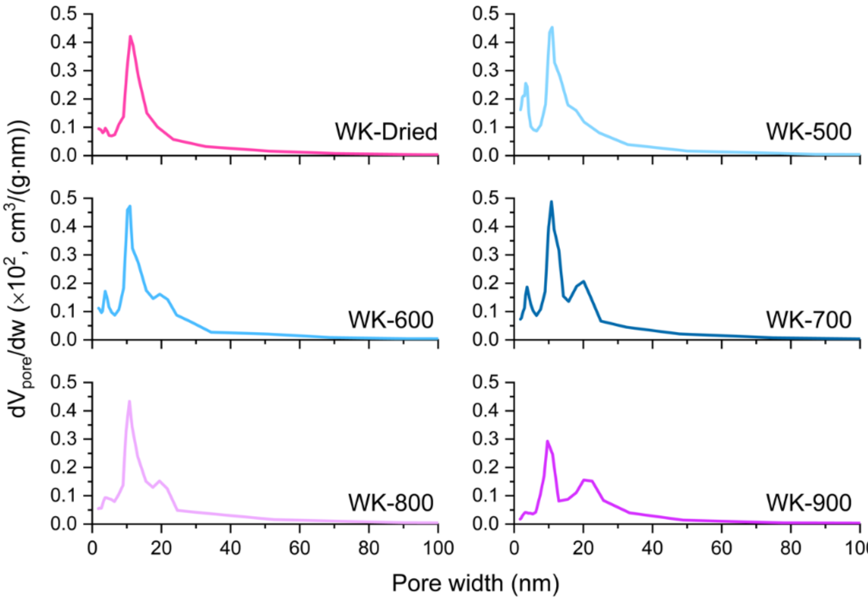

Figure S2. Pore size distribution of wet–kneaded samples. The distribution was calculated from N_2 desorption isotherms.

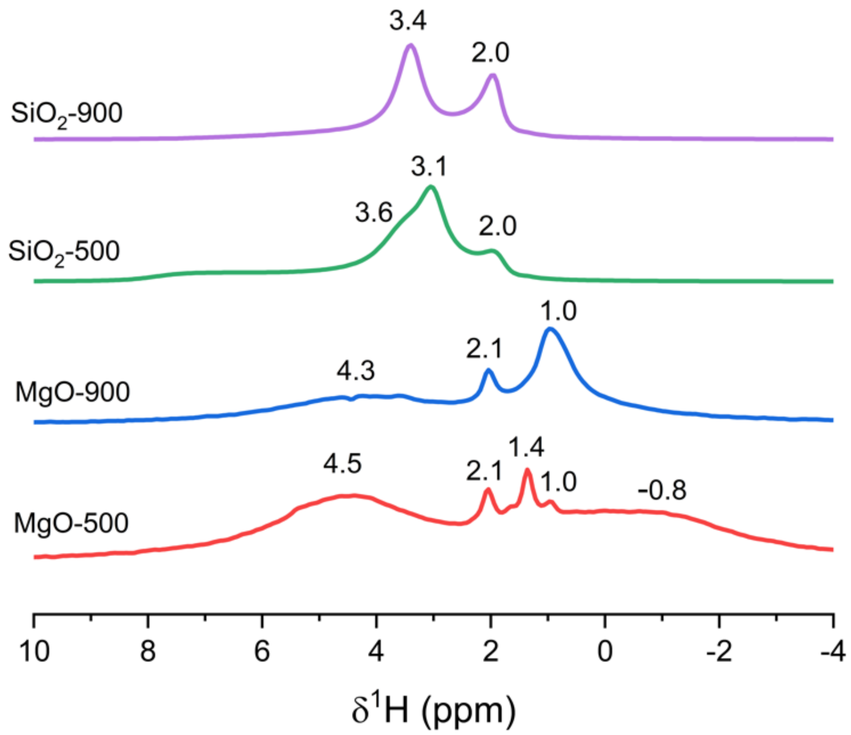

Figure S3. Solid–state ^{1}H spin–echo NMR spectra of wet–kneading precursors: SiO_2–900, SiO_2–500, MgO–900 and MgO–500. The numbers after sample name denotes the calcination temperature.

Table S1. Summary of the observed ^{29}Si species for wet-kneaded SiO_2–MgO catalysts by ^{1}H–^{29}Si CP MAS NMR.

Magnesium Silicates	WK-Dried	WK–500	WK–600	WK–700	WK–800	WK–900
Silica (Q4)	m	m	m	m	m	–
Silica (Q3)	s	s	s	s	m	–
Talc	s	s	s	m	w	–
Stevensite	s	m	m	m	m	s
Lizardite	m	m	m	s	s	–
Amorphous hydrous magnesium silicates	w	m	s	s	m	–
Enstatite	w	n.d.	n.d.	n.d.	n.d.	–
Intermediate between forsterite and enstatite	w	m	m	m	w	–
Forsterite	–	–	–	–	obs.	obs.

[1] The relative intensities of ^{29}Si species of each spectra by ^{1}H–^{29}Si CP MAS NMR: w (weak), m (medium) and s (strong). n.d. (not determined) due to the overlap with other ^{29}Si resonances; obs. (observed only by ^{29}Si DE MAS) It should be noted that the intensities of ^{29}Si species by ^{1}H–^{29}Si CP MAS NMR are not quantitative.

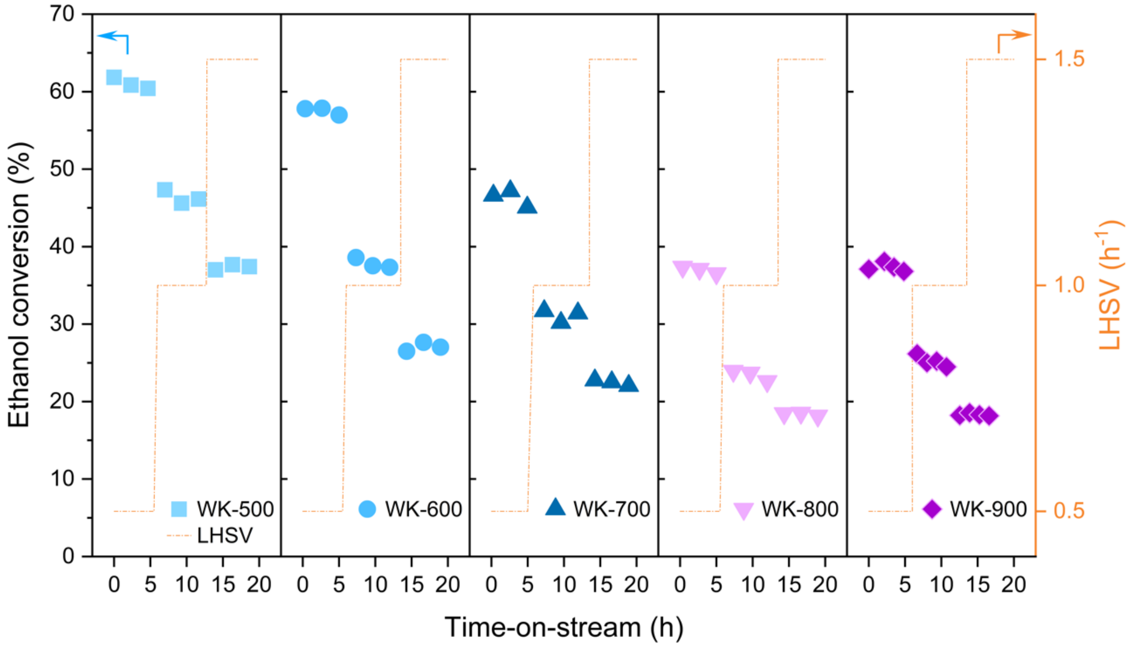

Figure S4. Detailed activity test results (ethanol conversion) of wet–kneaded SiO_2–MgO catalysts varying LHSV.

Table S2. Ethanol conversion and product selectivity of wet–kneaded SiO_2–MgO catalysts for Lebedev process varying LHSV. Acetaldehyde (AA), ethylene (C2=), 1,3–butadiene (butadiene), butenes (C4=) and C5+.

Catalyst	LHSV (h^{-1})	Ethanol Conversion (%)	Selectivity (%)				
			AA	C2=	Butadiene	C4=	C5+
Physical mixture of SiO_2–MgO	1.0	12.8	78.0	19.2	2.4	0.24	0.22
WK–500	0.5	60.8	19.9	33.5	36.1	4.2	6.1
	1.0	46.1	35.8	27.1	29.2	2.7	4.9
	1.5	37.0	41.6	25.1	25.9	2.0	5.2
WK–600	0.5	57.8	8.7	46.2	36.9	3.8	4.2
	1.0	37.3	22.4	41.2	31.8	2.8	1.6
	1.5	26.5	26.9	39.7	29.6	2.3	1.3
WK–700	0.5	45.0	7.1	52.7	35.2	3.3	1.5
	1.0	31.4	17.2	49.0	30.2	2.2	1.1
	1.5	22.7	20.3	48.9	27.7	1.8	1.0
WK–800	0.5	37.3	4.4	65.0	27.5	1.9	0.9
	1.0	28.6	9.6	61.3	26.9	1.4	0.6
	1.5	21.5	11.9	60.5	26.0	1.2	0.2
WK–900	0.5	37.1	32.2	27.9	34.0	3.5	2.9
	1.0	26.1	43.5	23.9	28.6	2.8	1.2
	1.5	18.2	44.9	26.4	25.5	2.5	0.6

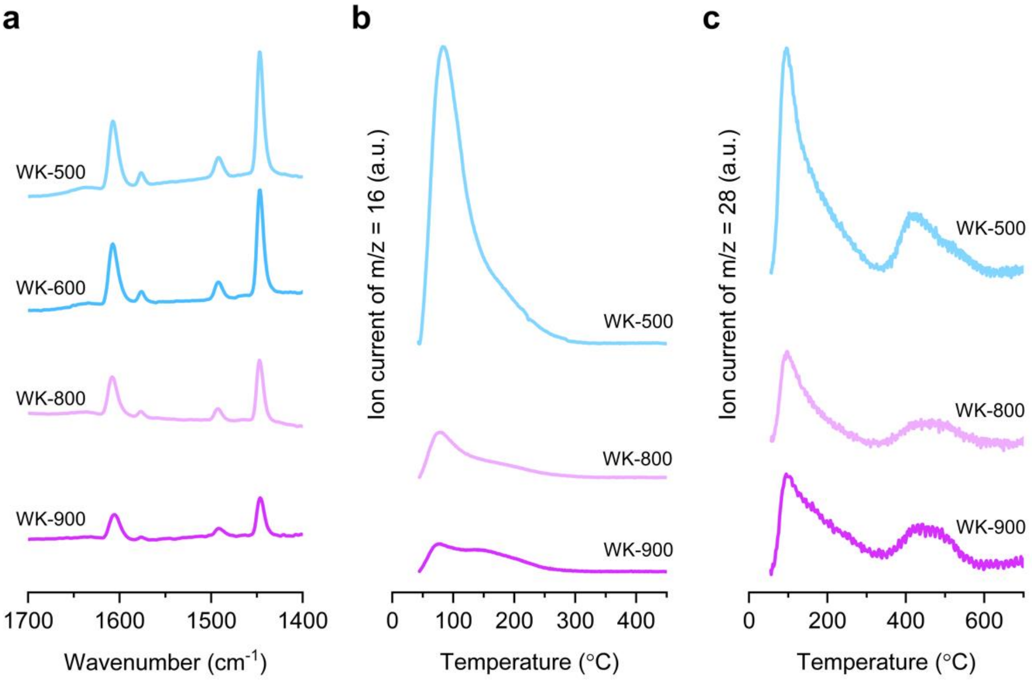

Figure S5. (**a**) FT–IR spectra of wet-kneaded SiO_2–MgO catalysts after adsorption of pyridine. Four bands at 1446, 1492, 1576 and 1607 cm^{-1} indicates the Lewis acid sites were only probed by pyridine-IR for wet-kneaded SiO_2–MgO catalysts. The spectra were taken after desorption of pyridine at 150 °C. The MS signals of (**b**) NH_3–TPD and (**c**) CO_2–TPD. The FT–IR spectra and MS signals of the NH_3– and CO_2–TPD were displayed with offset for clarity.

Table S3. Comparison of the ethanol conversion (C_{EtOH}), ethylene selectivity ($S_{ethylene}$), the rate of ethylene formation, the number of acidic and basic sites of wet-kneaded SiO_2–MgO catalysts characterized by pyridine–IR, NH_3–TPD and CO_2-TPD.

Catalyst	LHSV (h^{-1})	C_{EtOH} (%)	$S_{ethylene}$ (%)	Rate of Ethylene Formation ($mol_{ethylene}/g_{cat}/h$)	Number of Acidic Sites ($mmol/g_{cat}$)		Number of Basic Sites ($mmol/g_{cat}$)
					py–IR[1]	NH_3–TPD	CO_2-TPD
WK–500	1.5	37.0	25.1	3.0	0.39	0.50	0.20
WK–600	1.0	37.3	41.2	3.3	0.22	-	-
WK–700	1.0	31.4	49.0	3.3	-	-	-
WK–800	0.5	37.3	65.0	2.6	0.20	0.09	0.06
WK–900	0.5	37.0	27.5	1.1	0.20	0.08	0.10

[1] The number of acidic sites for pyridine–IR (py–IR) were calculated after the normalization of the spectra by the weight of the pellets and by the subtraction of the spectra obtained before pyridine adsorption, using the integral extinction coefficients of 0.96 cm/μmol^{-1} for Lewis acid site.[57].

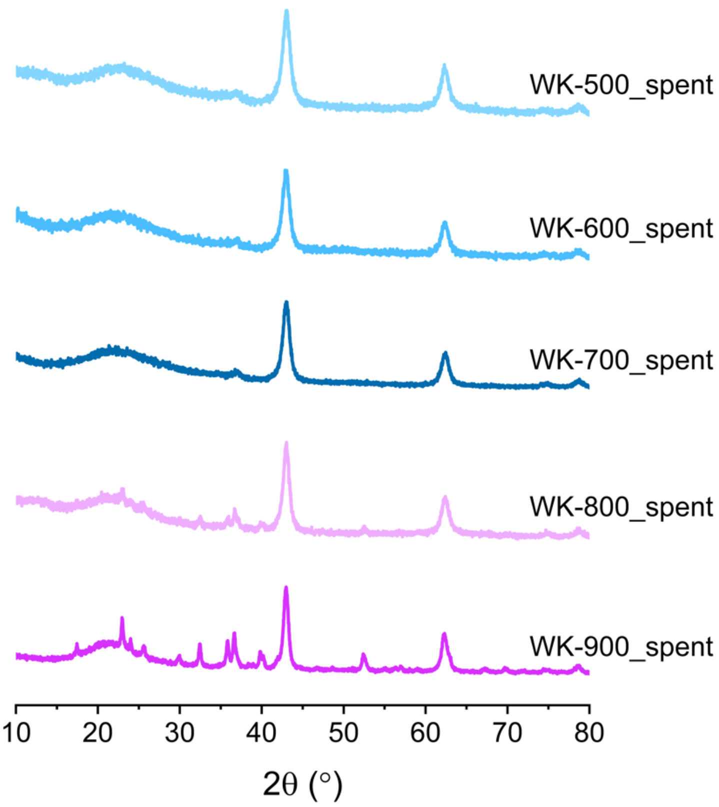

Figure S6. PXRD patterns of spent wet–kneaded SiO_2–MgO catalysts after reaction.

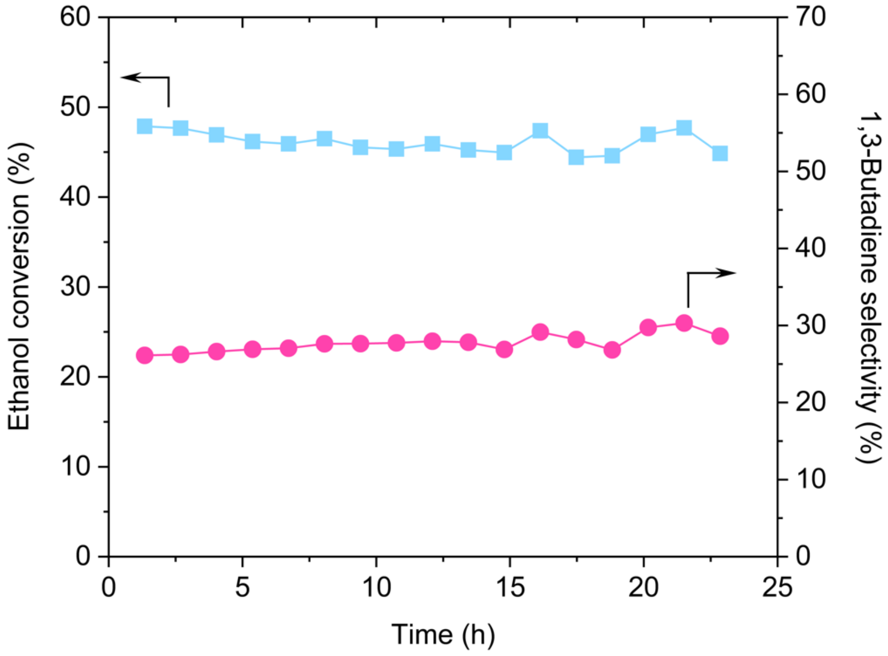

Figure S7. Ethanol conversion and 1,3–butadiene selectivity as a function of time–on–stream over WK–500 catalyst at LHSV 1.0 h^{-1}.
